# The emerging roles of miRNA-mediated autophagy in ovarian cancer

**DOI:** 10.1038/s41419-024-06677-8

**Published:** 2024-05-03

**Authors:** Yamin Ding, Xuan Huang, Tuo Ji, Cong Qi, Xuzhu Gao, Rongbin Wei

**Affiliations:** 1 Jiangsu Key Laboratory of Marine Pharmaceutical Compound Screening, College of Pharmacy, Jiangsu Ocean University, Lianyungang, China; 2 Institute of Clinical Oncology, The Second People’s Hospital of Lianyungang City (Cancer Hospital of Lianyungang), Lianyungang, China

**Keywords:** Cancer, Autophagy

## Abstract

Ovarian cancer is one of the common tumors of the female reproductive organs. It has a high mortality rate, is highly heterogeneous, and early detection and primary prevention are very complex. Autophagy is a cellular process in which cytoplasmic substrates are targeted for degradation in lysosomes through membrane structures called autophagosomes. The periodic elimination of damaged, aged, and redundant cellular molecules or organelles through the sequential translation between amino acids and proteins by two biological processes, protein synthesis, and autophagic protein degradation, helps maintain cellular homeostasis. A growing number of studies have found that autophagy plays a key regulatory role in ovarian cancer. Interestingly, microRNAs regulate gene expression at the posttranscriptional level and thus can regulate the development and progression of ovarian cancer through the regulation of autophagy in ovarian cancer. Certain miRNAs have recently emerged as important regulators of autophagy-related gene expression in cancer cells. Moreover, miRNA analysis studies have now identified a sea of aberrantly expressed miRNAs in ovarian cancer tissues that can affect autophagy in ovarian cancer cells. In addition, miRNAs in plasma and stromal cells in tumor patients can affect the expression of autophagy-related genes and can be used as biomarkers of ovarian cancer progression. This review focuses on the potential significance of miRNA-regulated autophagy in the diagnosis and treatment of ovarian cancer.

## Facts

- MiRNAs in ovarian cancer tissue can affect autophagy of ovarian cancer cells.
- MiRNAs regulate gene expression at the posttranscriptional level.
- MiRNA can serve as a biomarker for the progression of ovarian cancer.

## Open questions

- What mechanism causes abnormal miRNA expression?
- What are the mechanisms by which miRNAs influence ovarian cancer progression?
- How miRNAs mediate cellular autophagy?

## Introduction

Ovarian cancer (OC) is one of the common tumors of female reproductive organs, second only to cervical cancer and uterine corpus cancer in terms of incidence. However, deaths due to ovarian cancer are the first among all types of gynecologic tumors, posing a serious threat to women’s lives. It has been reported that 310,000 new cases of ovarian cancer are expected to be diagnosed each year and up to 200,000 patients die from it [1]. Due to the lack of early clinical manifestations of ovarian cancer, most patients are already in advanced stages when diagnosed, and radical surgery is difficult to perform, resulting in a high mortality rate of ovarian cancer [2]. For many cancers, the increase in survival rates is largely attributed to cutting-edge research and advances in screening, surgery, and treatment methods. However, ovarian cancer is characterized by significant tissue heterogeneity with genomic features, suggesting that it is susceptible to resistance to chemotherapy, leading to high rates of tumor recurrence [3]. Despite some advances in the treatment of ovarian cancer, the survival rate of ovarian cancer has not improved substantially for decades, even in resource-rich countries such as the United States and Canada. Therefore, there is an urgent need for new methods and tools to improve the diagnosis, prognosis, and treatment of ovarian cancer.

**Fig. 1:**
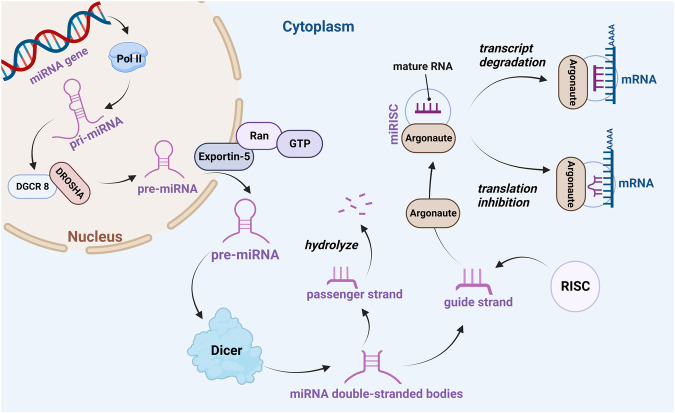
The miRNA biogenesis pathway. MiRNA genes are transcribed by RNA Pol II to produce pri-miRNAs. The pre-miRNA, is transported to the cytoplasm via the Exportin-5/RanGTP complex and is transported into the cytoplasm. Next, miRNA double-stranded bodies are produced by Dicer. After double-strand unwinding by the unwinding enzyme activity of Dicer, one strand will be degraded while the other strand is retained and eventually forms RISC. The miRISC containing the catalytic component of Argonaute proteins recognizes the complementary sequences that do not exactly match in its target and leads to translation inhibition or accelerated transcript degradation by uncapping and deadenylation.

In the last decades, microRNAs (miRNAs) have been shown to have therapeutic potential for a wide range of diseases [4]. miRNAs are a class of noncoding single-stranded RNAs encoded by endogenous genes, about 22 nucleotides in size, that play a role in almost all mammalian biological pathways, such as apoptosis, proliferation, regulation of cell cycle, and metabolism [5]. Each miRNA can have multiple target genes, while several miRNAs can also regulate the same gene, forming a complex regulatory network. Typically, miRNAs regulate gene expression by binding to the 3’-untranslated region (3’-UTR) of target mRNAs, controlling gene expression by targeting the 3’-UTR [6], 5’-UTR [7], and coding region [8] of specific mRNAs or by embedding in specific genes through posttranscriptional inactivation of target genes due to mRNA degradation or inhibition of translation [9]. miRNA expression may affect the extent of target regulation and thus cellular homeostasis. Disturbances in the internal environment of an organism are usually accompanied by abnormal synthesis or secretion of miRNAs in cells or blood. Therefore, many miRNAs have become recognized indicators of certain diseases, including Alzheimer’s disease [10], diabetes [11], viral infectious diseases [12], and cancer [13]. Studies have confirmed that aberrantly expressed miRNA are involved in the regulation of drug resistance in ovarian cancer, and upregulation of some miRNA expression can inhibit the development of drug-resistant cells in ovarian cancer [14]; while other miRNA overexpression can contribute to the development of drug-resistant cells in ovarian cancer [15]. The cellular biogenesis of miRNA is a multistep process in which most miRNA genes are transcribed by RNA polymerase II (Pol II) to produce pri-miRNAs with long primary transcripts. Pri-miRNAs are ~300–1000 bases in length with an incomplete matching stem-loop hairpin structure in the middle [16], and pri-miRNA is processed in the nucleus by a multiprotein complex called the microprocessor, whose core components are the RNase III enzyme DROSHA, the double-stranded RNA-binding protein DGCR8 and related proteins [17]. The product of DROSHA cleavage, a precursor miRNA of about 70 bases (pre-miRNA), is transported to the cytoplasm via the Exportin-5/RanGTP complex and is transported into the cytoplasm [18]. Next, miRNA double-stranded bodies are produced by another RNase III enzyme, Dicer. After double-strand unwinding by the unwinding enzyme activity of Dicer, one strand will be degraded while the other strand, also known as the guide strand (which becomes the mature form of miRNA) is retained and eventually forms a functional RNA-induced silencing complex (RISC) [19]. The miRNA-induced silencing complex (miRISC) containing the catalytic component of Argonaute (Ago) proteins recognizes the complementary sequences that do not exactly match in its target and leads to translation inhibition or accelerated transcript degradation by uncapping and deadenylation (Fig. 1).

**Fig. 2:**
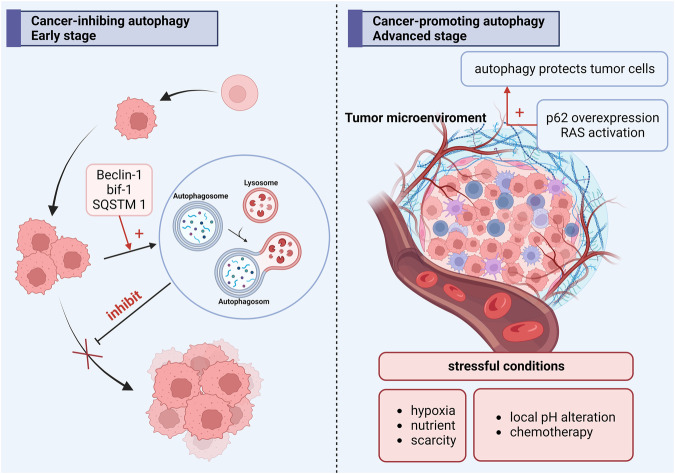
The dual role of autophagy in cancer. On the one hand, autophagy may play a role in limiting the earliest stages of tumorigenesis; on the other hand, autophagy can participate in reprogramming the cellular microenvironment and protect cancer cells from different survival stresses, thus favoring tumor progression.

Autophagy is an evolutionarily conserved intracellular catabolic degradation process that is the main pathway by which the body removes damaged, senescent, degraded, and nonfunctional proteins and organelles [20], and plays a key role in maintaining the balance between cell survival and death. It restores cellular homeostasis by providing materials and energy to synthesize new cellular components, thus allowing cells to resist survival stresses (e.g., nutrient deficiencies and hypoxia) [21]. There are three morphologically and mechanistically distinct types of autophagy in cells: macroautophagy, microautophagy, and chaperone-mediated autophagy [22], with macroautophagy usually referred to as autophagy [23]. Among the three types of autophagy, macroautophagy has the most typical and prevalent mechanism and is involved in the pathogenesis of the majority of autophagic dysfunctional diseases. In this paper, we focus our discussion on macroautophagy (hereafter referred to as autophagy), the main feature that distinguishes autophagy from the other two forms of autophagy is the formation of double-membrane structures called autophagosomes, which deliver damaged organelles and protein aggregates to lysosomes. Studies have shown that autophagy is closely associated with tumor development [24]; however, the role of autophagy in cancer is rather complex and remains somewhat controversial. On the one hand, autophagy may play a role in limiting the earliest stages of tumorigenesis; on the other hand, there is growing evidence that in diagnosed cancers, autophagy can participate in reprogramming the cellular microenvironment and protect cancer cells from different survival stresses (e.g., hypoxia, nutritional deficiency or cancer therapy) [25], thus favoring tumor progression. Cancer cells experience higher metabolic demands and stresses than normal cells because of their rapid proliferation and altered glycolytic metabolism and therefore may be more dependent on autophagy for survival [26]. Because of this, autophagy inhibition may improve the outcome of patients with advanced cancer. Furthermore, there is growing evidence that autophagy-mediated cell survival plays a key role in the etiology and progression of ovarian cancer (Fig. 2).

A growing number of studies published in the past few years have highlighted the importance of miRNAs in the regulation of autophagy. In this review article, we will briefly summarize miRNA and autophagic pathways, and analyze the link and correlation between autophagy, miRNA, and ovarian cancer.

## Molecular mechanisms of autophagy

The autophagic process involves a series of phases and sequential membrane remodeling events, including induction, vesicle nucleation, vesicle elongation, autophagosome maturation and lysosome fusion, degradation, recycling and autophagic lysosome reformation. Studies have shown that miRNAs are key regulators of the autophagic process and are involved in several steps of autophagy, and also that autophagy is critical in the production and maintenance of miRNAs.

### Induction

In mammalian cells, autophagy induction is initiated by activation of the ULK complex, which consists of ULK1 or ULK2, ATG13, FIP200, and ATG101 [27], which acts directly on downstream mTORC1. mTORC1 is a key negative autophagy regulatory complex that phosphorylates ULK1 under nutrient-rich conditions and mammalian ATG13 (mATG13), leading to inhibition of the activity of the complex and blocking autophagy activation. However, under starvation conditions, inactive mTOR allows phosphorylation of ULK1 itself, mATG13, and FIP200, leading to further recruitment of the ATG complex to initiate autophagy [28]. As a major upstream regulator of the autophagic pathway, the mTOR-containing protein complexes and other proteins in this pathway are direct or indirect targets of many miRNAs.

### Vesicle nucleation

The second step in autophagy is vesicle nucleation. This is the process of recruiting proteins and lipids to build the autophagosomal membrane. This process is initiated by the activation of the PI3K complex, which consists of the PI3K proteins VPS34 and VPS30, ATG14/Barkor, VPS15, and ATG6/BECN1 (Beclin1) proteins [29]. the lipid kinase activity of the PI3K complex is derived from the accumulation of phosphatidylinositol 3-phosphate (PI3P) molecules in the membrane, including the PI3P molecules that act as binding sites for autophagy-associated proteins (e.g., WIPI1-4 and DFCP1), mark sites for autophagosome formation and lead to the production of omegasome/cradle.

### Vesicle elongation

Two unique ubiquitin-like coupling systems are involved in vesicle amplification. In the first system, the ubiquitin-like protein ATG12 is covalently coupled to ATG5 in the presence of the E1-like enzyme ATG7 and the E2-like enzyme ATG10. The ATG12-ATG5 coupling then localizes to the autophagosomal membrane and forms a large multimeric complex with ATG16L1, which promotes the amplification of the autophagosomal membrane [30]. The second system involves the coupling of LC3 to phosphatidylethanolamine (PE), which should be cleaved by ATG4 cysteine protease at its carboxyl terminus to produce a cytoplasm-free LC3-I form capable of lipid binding [31]. The E1-like enzyme ATG7, E2-like enzyme ATG3, and E3-like enzyme ATG12-ATG5-ATG16L1 are required for PE coupling to free LC3-I protein coupling required to produce the autophagic membrane-bound LC3-II form. In this way, LC3 proteins ensure the elongation and expansion of the autophagic membrane and its closure.

### Autophagosome maturation and lysosome fusion

The final key step in the autophagic process is the fusion of autophagosomes with lysosomes to form autolysosomes [32]. Autophagosomes undergo several independent fusions to form amphiphiles with late endosomes and subsequently form autolysosomes with lysosomes [33]. This process requires membrane fusion mechanisms, including SNARE complexes (e.g., VAMP8, STX17), RAB proteins (e.g., RAB5 and RAB7), and integral lysosomal proteins (e.g., LAMP2). BIF-1 and UVRAG proteins play a role in regulating membrane curvature formation and endosome transport, thereby facilitating autophagosome formation through their interaction with Beclin1 proteins. maturation of autolysosomes through their interaction with Beclin1 proteins. After fusion, autolysosomes carry redundant or dysfunctional organelles and misfolded proteins that are degraded by lysosomal acid hydrolases (including histones) for recycling and metabolism in the substrate.

### Degradation, recycling, and autophagic lysosome reformation

Substance degradation and catabolic product efflux. The degradation step of autophagy is accomplished through acid hydrolases in vesicles/lysosomes, and these extensive hydrolases allow efficient breakdown and recycling of cellular components delivered through autophagy, with the resulting “building blocks” (e.g., amino acids) being released back into the cytoplasm by permeases for reuse by the cell in anabolic or catabolic pathways [34]. During the process of autophagy, a single autophagosome can fuse with multiple lysosomes, and as autophagy proceeds, lysosomes may become depleted. Therefore, maintaining the dynamic balance of lysosomes is crucial [35]. Research has revealed that the late stages of autophagy involve a process known as autophagic lysosome reformation (ALR), which entails the regeneration of functional lysosomes from autolysosomes [36]. During the ALR process, buds form on the autolysosome membrane, which then extrude along microtubules to generate tubules known as “reformation tubules.” The severance of these reformation tubules yields membrane fragments (“proto-lysosomes”), which mature into functional lysosomes, subsequently re-fusing with autophagosomes to participate in autophagy, thus maintaining the dynamic equilibrium of free lysosomes within the cell [37].

## Interrelation between miRNAs and autophagy

It has been shown that miRNAs are new players in the regulation of autophagy and are involved in various steps of autophagy, ranging from the upstream signaling pathway to the later stages of autolysosomal degradation. On the other hand, autophagy is also involved in the regulation of miRNA expression and homeostasis in vivo [38, 39].

### Regulation of autophagy by miRNAs

**Fig. 3:**
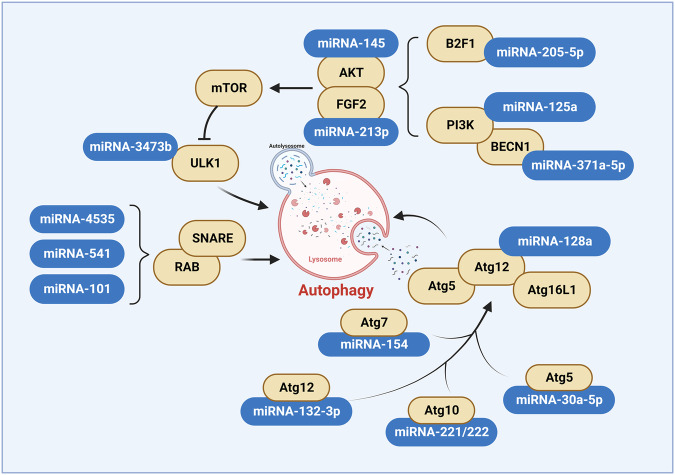
Multiple signaling pathways are implicated in the regulation of autophagy by miRNA. Blue represents miRNAs involved in autophagy regulation, and yellow indicates the targets of miRNA.

Of ~3000 miRNAs reported to date, hundreds are directly involved in autophagy regulation. Genes associated with proteins that act at different stages of the autophagic pathway have been shown to have targets for a large number of miRNAs (Fig. 3). These miRNAs can regulate autophagy by modulating various stages of the autophagic process.

#### The regulation of induction phase by miRNAs

Many miRNAs have been shown to regulate the induction phase of autophagy. Genes for mTOR, a central regulator of autophagy, were identified as targets of several miRNAs associated with autophagy, including miRNA-145 [40], miRNA-375 [41], miRNA-125a [42], miRNA-21-3p [43], miRNA-3473b [44]. other components of the mTOR complex, such as mLST8, DEPTOR, Tti/Tel2, RAPTOR, RICTOR, PRAS40, and mSIN1 are regulated by different groups of miRNAs. mTOR regulators RHEB, TSC1/2, and RAG have also been identified as targets of miRNAs in the context of autophagy. Other related signaling pathways are no exception. miRNAs regulate the abundance and activity of AKT pathway components, including IGFR1, IP3K2, PIK3CD, PTEN, and AKT. cellular energy sensors LKB1 and AMPK and calcium sensor CaMKKb are also under miRNA control.

For example, Yan et al, explored the effect of miRNA-145 on acute myocardial infarction (AMI) and its potential mechanisms. The results from rat experiments showed that miRNA-145 inhibited myocardial infarction-induced apoptosis through autophagy associated with the AKT3/mTOR signaling pathway in vivo and in vitro [40]. Chen et al, explored the potential function and potential mechanisms of miRNA-125a in thyroiditis. It was found that miRNA-125a inhibited autophagy via PI3K/AKT/mTOR signaling pathway in a model of thyroiditis [42].

Lizhu Ma et al. used bovine ovarian granulosa cells (BGCs) as a model to elucidate the autophagy and role of miRNA-21-3p in bovine ovaries. It was shown that miRNA-21-3p targets FGF2 and inhibits autophagy in BGCs by suppressing AKT/mTOR signaling [43]. Moreover, miRNA-3473b may serve as a potential target to regulate the role of autophagy in the pathogenesis of inflammation by targeting TREM2/ULK1 expression to regulate the secretion of pro-inflammatory mediators [44].

#### The regulation of vesicle nucleation phase by miRNAs

Vesicle nucleation is a critical step in assembling the autophagosomal membrane by recruiting proteins and lipids. In mammalian cells, this process is primarily initiated by the activation of the PI3K/Beclin1 complex, which includes core members hVPS34, Beclin1, and p150. Many other associated proteins of this complex also act as regulatory factors, including BAX-1, ATG14L, UVRAG, Ambra1, and Rubicon.

miRNA-205-5p targets E2F1, thereby inhibiting SKP2-mediated Beclin1 ubiquitination to promote macrophage autophagy and suppressing silicosis in mice with pulmonary fibrosis [45]. miRNA-371a-5p enhances the response of oxaliplatin to hepatic malignancies by targeting the inhibition of Beclin1-dependent autophagy [46]. miRNA-375 targets ATG14 to inhibit autophagy and sensitize hepatocellular carcinoma cells to sorafenib [41].

#### The regulation of the vesicle elongation phase by miRNAs

During the process of Vesicle Elongation, components of autophagy-related ubiquitination have also been shown to be regulated by miRNAs. miRNA-128a was found to inhibit chondrocyte autophagy and exacerbate knee osteoarthritis by disrupting ATG12 [47]. miRNA-30a-5p inhibits lung squamous cell carcinoma through ATG5-mediated autophagy [48]. miRNA-30a-5p inhibits cellular autophagy and promotes vein graft restenosis by targeting ATG5 [49]. miRNA-142-3p inhibits Mycobacterium tuberculosis-induced autophagic activation by targeting ATG16L1 and ATG4c [50]. Dan Hong injection inhibits autophagy through the miRNA–132-3p/ATG12 signaling axis and alleviates cerebral ischemia–reperfusion injury [51].

Junfeng Zhang et al. observed that overexpression of miRNA-154 inhibited bladder cancer cell growth and suppressed ATG7 expression in vivo, suggesting that miRNA-154 may function as a tumor suppressor in bladder cancer and that miRNA-154 may be a potential therapeutic target for bladder cancer patients [52].

Shen et al. found that miRNA-221/222 decreased ATG10 mRNA and protein levels, and silencing ATG10 significantly eliminated the effects of miRNA-221/222 on apoptosis and autophagy, thus suggesting that miRNA-221/222 can promote migration and invasion and inhibit autophagy and apoptosis by targeting ATG10 in thyroid tumor cells [53].

miRNAs have been reported to regulate the maturation steps of autophagy. Zhang et al. revealed the role of miRNA-101 in regulating autophagy through multiple targets, including RAB5A [54].

#### The regulation of autophagosome maturation and lysosome fusion phase by miRNAs

Many miRNAs also control the mechanism of autophagosome-lysosome fusion. RAB1B1 was described as another miRNA target. In hepatocellular carcinoma cells, miRNA-541 suppressed the malignant phenotype and autophagy of hepatocellular carcinoma cells by inhibiting ATG2A or RAB1B [55]. Similarly, RAB27A levels depend on miRNA-4535 [56].

#### The regulation of autophagic lysosome reformation phase by miRNAs

It has been reported that during prolonged starvation, the activation of mTORC1 promotes autophagic flux by inducing the reformation of autolysosomes, thus maintaining lysosomal balance [57]. Various miRNAs such as miRNA-302-367, miRNA-365, miRNA-4645-5p, have been shown to regulate autophagy by modulating mTORC1 activity [58–60]. The number of miRNAs that regulate autophagy has been found to be increasing.

### Regulation of miRNAs by autophagy

To date, there have been many studies on miRNA regulation of autophagy. However, recent studies have demonstrated evidence of mutual control between the autophagy pathway and the miRNA machinery. Indeed, in some other cases, degradation mediated through autophagy and autophagy can target miRNA and miRNA machinery components thus playing an important role in the control of cancer progression [61–63].

Autophagy is thought to play an important role in the maintenance of intracellular homeostasis. Gibbings et al. reported that NDP52-mediated autophagy is able to selectively degrade the miRNA processing enzyme DICER and the major miRNA effector AGO. DICER and AGO 2 levels are reduced by serum starvation or by activation of autophagy with an mTOR inhibitor (rapamycin (RAP)) [64]. Interestingly, miRNAs can also be directly degraded during autophagy; for example, Lan et al. demonstrated that mature miRNA-224 was selectively degraded by the autophagosome-lysosome system and that downregulation of autophagy was negatively correlated with miRNA-224 expression in hepatitis B virus (HBV) associated HCC specimens [65]. In another study, Peng et al. found that autophagy promoted through CDKN1B was able to selectively degrade miRNA-6981 by using the autophagy inhibitor bafilomycin (BAF) [66].

## Roles of autophagy-related miRNAs in ovarian cancer

**Fig. 4:**
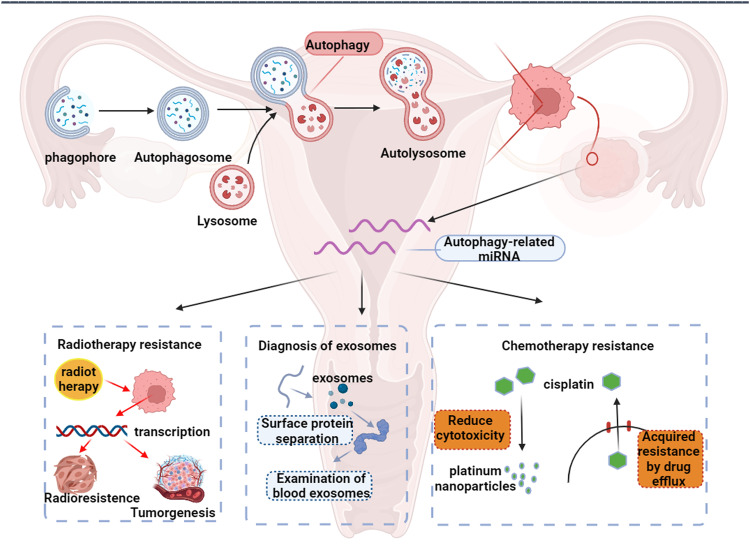
Roles of autophagy-related miRNAs in ovarian cancer. miRNAs produce therapeutic effects on ovarian cancer and reversal of antitumor drug cisplatin resistance through regulation of autophagy. The modulation of ovarian cancer cell sensitivity to radiotherapy through miRNA-mediated regulation of autophagy. miRNAs’ expression patterns are closely related to the clinicopathological characteristics of tumors. miRNAs have the potential to become new targets for tumor diagnosis and treatment.

With the increasing understanding of the regulatory role of miRNAs in autophagy and tumor development, the potential of miRNAs in tumor therapy and diagnosis has received increasing attention (Fig. 4). Currently, there exists a large body of literature reporting on the important role of miRNAs in regulating ovarian cancer autophagy [67], as shown in Table 1. which produces therapeutic effects on ovarian cancer and reversal of antitumor drug cisplatin resistance through regulation of autophagy. For example, Li et al. demonstrated that miRNA-20a-5p inhibited autophagy and cisplatin resistance in ovarian cancer cells through DNMT3b-mediated methylation of RBP1. It was shown that miRNA-20a-5p plays an important role in the regulation of autophagy and cisplatin resistance in ovarian cancer cells [68]. On the other hand, there are also studies reporting the modulation of ovarian cancer cell sensitivity to radiotherapy through miRNA-mediated regulation of autophagy [69]. miRNAs’ expression patterns are closely related to the clinicopathological characteristics of tumors. A study investigated 31 ovarian cancer patients who underwent miRNA sequencing and validated the sequencing results in multiple independent data sets. The results showed that the selected miRNAs had high diagnostic accuracy (AUC = 0.99) in patients with stage I high-grade plasmacytotic ovarian cancer [70]. Thus, miRNAs have the potential to become new targets for tumor diagnosis and treatment.

**Table 1:** Autophagy-associated miRNAs in ovarian cancer.

Effect on autophagy	Name	Dysregulation	Target/mechanism	Types of cancer cell line	Ref.
Activation	miRNA-378d	Upregulated	–	KGN	[126]
Activation	miRNA-342-3p	Upregulated	Enhanced Beclin1 ubiquitination and degradation	SKOV3 and OVCAR3	[127]
Activation	miRNA-30d-5p	Upregulated	–	PEO-1 and A2780	[128]
Activation	miRNA-29b-3p	Downregulated	–	A2780/paclitaxel	[129]
Inhibition	miRNA-152	Downregulated	Reduced ATG14 protein expression	A2780/CP70 and SKOV3/DDP	[130]
Inhibition	miRNA-8485	Downregulated	Decreased LAMTOR3 and mTOR expression while increasing ATG13 and LC3-II expression	SKOV3	[77]
Inhibition	miRNA-30a	Upregulated	–	A2780 and A2780/DDP	[115]
Inhibition	miRNA-20a-5p	Upregulated	DNMT3B-induced RBP1 DNA methylation.	A2780, COC1, A2780/DDP and COC1/DDP	[67]
Inhibition	miRNA-125b	Upregulated	Decreased LC3-II expression	KOV3, COC1 and COC1/DDP	[118]
Inhibition	miRNA-1305	Downregulated	The BECLIN1 and VPS34 binding to ARH-I is decreased	OAW42, OVCAR3 and KURAMOCHI	[131]
Inhibition	miRNA-3127-5p	Upregulated	–	SKOV3, A2780, SKOV3/paclitaxel and A2780/paclitaxel	[78]
Inhibition	miRNA-133a	Downregulated	Reduced YES1 expression	A2780/DDP and SKOV3/DDP	[116]
Inhibition	miRNA-219-5p	Downregulated	Decreased LC3-II expression	HEY, HO8910PM, HO8910, SKOV3, SKOV3/DDP, A2780 and A2780/DDP	[79]
Inhibition	miRNA-4478	Downregulated	Decrease LC3-II/LC3-I levels and increase p62 levels	IOSE80 and SKOV3	[69]
Inhibition	miRNA-506-3p	Downregulated	–	HOSE, OC, SKOV3, A2780, and HO8910	[76]
Inhibition	miRNA-144-3p	Downregulated	Decrease Beclin1 and ATG5 expression and increase p62 expression	SKOV3 and A2780	[75]
Inhibition	miRNA-214	Upregulated	–	SKOV3 and IOSE80	[105]
Inhibition	miRNA-1251-5p	Upregulated	Autophagosomes were greatly reduced	A2780, HEY, SKOV3, OVCA420, OVCA429, and OVCA433	[132]
Inhibition	miRNA-1301	Upregulated	Inhibited ATG5 and Beclin1 expression	SKOV3 and SKOV3/DDP	[133]
Inhibition	miRNA-199a-5p	Downregulated	Suppressed autophagy via GSK3β/mTOR complex signaling	SKOV3	[134]
Inhibition	miRNA-29c-3p	Downregulated	Inhibited autophagy by downregulating the FOXP1/ATG14 pathway	SKOV3/DDP and A2780/DDP	[135]

### Cellular context-dependent functions of miRNAs in ovarian cancer

miRNAs, as a group of endogenous small single-stranded noncoding RNA molecules consisting of ~22 nucleotides, regulate gene expression at the posttranscriptional level. miRNA expression is affected by amplification, deletion or mutation. These processes are controlled in tumor cells by tumor-inducing and tumor suppressor genes, which regulate different mechanisms of cancer development and progression, including tumor cell proliferation, cell growth, apoptosis, DNA repair, invasion, angiogenesis, metastasis, drug resistance, metabolic regulation, and modulation of immune responses in tumor cells [71, 72].

miRNAs may show different expression patterns due to specific tissues or different differentiation states, and a single miRNA can regulate multiple targets, so some miRNAs may show opposite roles in different environments. For instance, in ovarian cancer, KRAS demonstrates oncogenic properties, whereas miRNA-let-7d exerts anticancer effects by attenuating KRAS through HMGA2 suppression [73]. However, another study comparing epithelial ovarian cancer cell lines and immortalized ovarian surface epithelial cell lines reported a significant reduction in miRNA-let-7d expression in cancer cells, suggesting potential cellular or environment-dependent variability in miRNA-let-7d expression within the tumor entity [74].

The expression of miRNAs is highly specific to cell types and developmental stages. Consequently, the occurrence of aberrant miRNA expression is prevalent in ovarian cancer and is associated with disease progression. Numerous miRNAs have been identified to exhibit differential expression in ovarian cancer. These variations in miRNA abundance are typically associated with changes in cell migration, invasion, and metastasis. Compared to normal ovarian tissue, miRNA-144-3p [75], miRNA-4478 [69], and miRNA-506-3p [76] are upregulated in ovarian cancer tumors, whereas miRNA-8485 [77], miRNA-3127-5p [78], and miRNA-219-5p [79] are downregulated. However, there are also conflicting research findings. For instance, miRNA-23a, miRNA-552, miRNA-509-3p, and miRNA-19a show differential expression in ovarian cancer samples, with both upregulation and downregulation observed [80–83]. These findings suggest that miRNAs may exhibit functional variability in different cellular environments, necessitating further investigation into their underlying mechanisms.

### Dual roles of autophagy in cancer

Autophagy is essentially a protective pathway for eukaryotic cells to remove damaged organelles and misfolded proteins via lysosomes. Autophagy plays a dual role at different stages during cancer development and progression.

On the one hand, autophagy plays a suppressive role in the early stages of tumorigenesis. For example, Beclin1 is a protein required for autophagy induction, and its characterization has shown that autophagy is closely associated with human cancers. Beclin1, a haploinsufficient tumor suppressor gene, has been found to be deficient in breast, ovarian, and prostate cancers [84–87]. Higher spontaneous frequencies of hepatocellular carcinoma, lung cancer, and lymphoma have also been reported in Beclin1^+/−^ mice [84, 88]. Another key autophagy gene, Bif-1, also has a tumor suppressor role. bif-1 is able to interact with and positively regulate autophagy through UV radiation resistance-related genes, and Beclin1 depletion inhibits autophagy formation and leads to tumor cell proliferation [89, 90]. p62 protein, also known as SQSTM1, has as one of its main functions the recruitment of proteins into aggregates for autophagic degradation. p62 overexpression activates DNA damage response and endoplasmic reticulum stress to enhance tumor growth, which is protective of cell survival. p62 accumulation results from autophagy inhibition [91], whereas p62 lowering contributes to certain diseases by disrupting autophagic degradation. Ai et al., in their study of the effect of miRNA-107 on autophagy, proliferation, and migration of breast cancer cells, found that in the case of MDA-induced proliferation and migration of breast cancer cells, p62 was not detected. Ai et al. in studying the effects of miRNA-107 on autophagy, proliferation, and migration of breast cancer cells, found that overexpression of miRNA-107 in MDA-MB-231 and MDA-MB-453 cells resulted in upregulation of p62 and downregulation of Beclin1, leading to a decrease in the ability of cells to proliferate and migrate [92].

On the other hand, when the tumor progresses to an advanced stage, autophagy protects the viability of tumor cells under stressful conditions (e.g., hypoxia, nutrient scarcity, local pH alteration, and chemotherapy), which provides favorable conditions for the growth and proliferation of tumor cells [93]. For example, RAS activation can lead to the upregulation of autophagy, which in turn plays an enhanced role in the growth and survival of tumor cells [94].

### Involvement of miRNAs and autophagy in cancer biology

Many miRNAs with regulatory effects on autophagy are involved in many biological functions of cancer, including tumor cell proliferation, metabolism, migration, metastasis, tumor tissue angiogenesis, and treatment of cancer. Moreover, some autophagy-associated miRNAs have been considered as cancer biomarkers or studied as anticancer targets, as shown in Table 2. Therefore, this section summarizes the impact of some autophagy-associated miRNAs on tumor biology and their applications in cancer therapy.

**Table 2:** Involvement of miRNAs and autophagy in cancer biology.

Effect on autophagy	Name	miRNA expression regulation	miRNA functions	Tested cell line (tissue origin)	Ref.
Inhibition	miRNA-200c	Upregulated	Inhibition of cell proliferation	SKOV3/Olaparib	[136]
Inhibition	miRNA-125a-3p	Upregulated	Inhibition of cell proliferation, migration, and invasion, induction of cellular senescence	SKOV3 and A2780	[137]
Inhibition	miRNA-1305	Downregulated	Inhibition of cell proliferation and tumor growth	Ovarian tumors	[138]
Inhibition	miRNA-194-5p	Upregulated	Inhibition of cell viability, promotion of apoptosis	A2780/DDP and SKOV3/DDP	[139]
Inhibition	miRNA-141-3p	Downregulated	Inhibition of cell proliferation, migration, and invasion	SKOV3 and A2780	[140]
Activation	miRNA-144	Downregulated	Enhancement of cell viability	KGN	[141]
Inhibition	miRNA-30b-5p	Upregulated	Inhibition of cell proliferation, promotion of apoptosis	Ovarian granulosa cell	[142]
Inhibition	miRNA-21	Upregulated	Inhibition of cell growth, induction of apoptosis	AGS/DDP	[143]
Inhibition	miRNA-486	Upregulated	Inhibition of cell viability and proliferation, induction of apoptosis	Ovarian granulosa cell	[144]
Inhibition	miRNA-29c‐3p	Upregulated	Inhibition of cell migration and invasion	SKOV3	[145]
Inhibition	miRNA-let-7b/7c-5p	Upregulated	Inhibition of tumor growth	HEY and SKOV3	[146]
Inhibition	miRNA-654	Upregulated	Inhibition of cell survival, induction of apoptosis	Mouse OGCs	[147]
Inhibition	miRNA-29c-3p	Upregulated	Inhibition of cell proliferation	SKOV3/DDP A2780/DDP	[135]
Inhibition	miRNA-199a	Downregulate	Inhibition of cell proliferation and invasion	SKOV3/DDP	[134]
Inhibition	miRNA-1301	Upregulated	Inhibition of cell proliferation and invasion	SKOV3/DDP	[133]
Inhibition	miRNA-506-3p	Upregulated	Inhibition of cell proliferation, promotion of apoptosis	SKOV3/CBP and A2780/CBP	[76]
Inhibition	miRNA-219-5p	Upregulated	Inhibition of cell proliferation, induction of apoptosis	A2780/DDP and SKOV3/DDP	[79]
Inhibition	miRNA-3127-5p	Downregulated	Inhibition of cell viability, migration, and invasion	SKOV3, A2780, SKOV3/PTX and A2780/PTX	[78]
Inhibition	miRNA-125b	Upregulated	Inhibition of cell proliferation, induction of apoptosis	COC1/DDP	[118]
Inhibition	miRNA-29b-3p	Downregulate	Inhibition of cell viability and proliferation	SKOV3/PTX and A2780/PTX	[129]
Inhibition	miRNA-4478	Upregulated	Inhibition of cell proliferation	ES-2 and SKOV3	[69]

#### Cell survival and proliferation

Many miRNAs have been shown to regulate autophagy, thereby exerting crucial effects on cancer cell growth and proliferation. For example, it was found that overexpression of miRNA-99b increased the expression of autophagy-associated biomarkers, and miRNA-99b induced autophagy and thus inhibited the growth and proliferation of prostate cancer cells by targeting mTOR and AR signaling [95]. miRNA-4478 was maintained at low levels in ovarian cancer, and in ovarian cancer cells exposed to radiation, miRNA-4478 expression decreases over time, which in turn causes poor prognosis in ovarian cancer [69], but when miRNA-4478 is upregulated, it can delay the proliferation of ovarian cancer cells and sensitize them to radiation. Yihan Wang et al. also found that overexpression of miRNA-8485 inhibited the proliferation and promoted apoptosis in SKOV3 cells by CCK-8 assay and flow cytometry analysis [77]. Aerobic glycolysis is significantly upregulated in pancreatic cancer cells. miRNA-7 further affects the growth and proliferation of pancreatic cancer cells by directly targeting autophagy-related genes, including LKB1, ULK2, ATG 4A, and ATG7 and thereby regulating the autophagy-induced and vesicle elongation phases in order to reduce the intracellular supply of glucose to aerobic glycolysis [96].

#### Cell metabolism

Cancer is usually accompanied by dramatic changes in tumor cell metabolism, and autophagy is one of the cellular functions related to metabolism. Therefore, autophagy production is often correlated with tumor cell metabolism. Dysregulation of autophagy also affects the imbalance of metabolic homeostasis and leads to a variety of diseases. miRNA-let-7 can then regulate autophagy by mediating the metabolic changes of glycolysis, which in turn regulates cancer progression [97]. miRNA-7 can inhibit autophagy through the upregulation of LKB1-AMPK-mTOR signaling, and directly target autophagy induction and vesicle elongation stages to reduce intracellular glucose supply for glycolytic metabolism and impair autophagy-derived glucose pools to inhibit pancreatic cancer progression [96]. Da-Hye Lee et al. found that inhibition of miRNA-214-3p expression ameliorated fatty liver disease by increasing autophagic activity through increased ULK1 expression. Therefore, it is believed that miRNA-214-3p is a potential therapeutic target for nonalcoholic steatosis [98]. A large number of studies have shown that miRNAs can participate in the regulation of autophagy in tumor cells through the regulation of cellular metabolism, which in turn affects cancer progression. However, the specific regulatory network is intricate and complex and needs to be analyzed by more in-depth studies.

#### Angiogenesis

Autophagy is important for endothelial cell function and angiogenesis. Several autophagy-regulating miRNAs have been shown to influence endothelial cell survival, growth, and proliferation, thereby directly affecting tumor angiogenesis. For example, miRNA-212, which is downregulated in prostate cancer, is able to negatively regulate autophagy by targeting the autophagy activator SIRT1 [99]. Under these conditions, angiogenesis is inhibited, and cellular senescence occurs in prostate cancer cells. Inhibition of miRNA-195 targeting the autophagy protein GABARAPL1 promotes proliferation, migration and angiogenesis of endothelial progenitor cells (EPCs) under hypoxic conditions [100]. In addition, it was shown that lncRNA ANRIL intervening in the autophagy pathway through miRNA-99a and miRNA-449a was able to promote angiogenesis [101]. In another study, lncRNA WTAPP1 acted as a molecular decoy for miRNA-3120-5p to regulate MMP-3 expression through the PI1K/Akt and autophagy pathways, thereby mediating cell migration and angiogenesis in endothelial progenitor cells [102].

The above findings demonstrate the importance of miRNAs and autophagy in regulating endothelial cell homeostasis and tumor angiogenesis in vitro, and their correlation with tumor angiogenesis in vivo needs to be determined by further studies.

#### Cancer cell migration and metastasis

Cell motility, invasion and metastatic spread of tumor cells are also affected by the link that exists between cellular autophagy and migration, and some miRNAs regulating autophagy are also associated with cancer migration and metastasis.

In some studies, miRNAs were able to influence cancer cell migration and metastasis by negatively regulating autophagy. For example. Song et al. found that miRNA-219-5p attenuates cisplatin resistance of ovarian cancer by inactivating Wnt/β-catenin signaling and autophagy via targeting HMGA2 and significantly inhibited tumor cell proliferation and migration [79].

In some instances, miRNA-activated autophagy regulates cancer migration and metastasis. For example, nutrient depletion and rVP1 (recombinant foot-and-mouth disease virus capsid protein) -mediated autophagy was able to up-regulate miRNA-let7a-3p thereby inhibiting ovarian cancer cell migration. This result was obtained by analyzing whether miRNA-let7a-3p mimics inhibit MMPs (matrix metalloproteinases), a key mediator of ovarian cancer cell migration and invasion [103].

## Autophagy-related miRNAs and their response to ovarian cancer therapy

### Response to radiotherapy

Radiation therapy can be used as a local treatment for ovarian cancer, and its mechanism is to inhibit and kill tumor cells through radiation ionization [104]. However, radiation-insensitive or radiation-resistant ovarian cancer cells are often encountered in the actual treatment process, which limits the application of radiation therapy in ovarian cancer. Recent studies have shown that miRNAs can regulate the sensitivity of ovarian cancer cells to radiotherapy. For example, in radiation-exposed ovarian cancer cells, downregulation of miRNA-4478 expression over time led to poor prognosis of ovarian cancer patients. But upregulation of miRNA-4478 could be targeted and inhibited fused in sarcoma (Fus). Fus was upregulated in ovarian cancer, and its expression was further elevated in irradiated ovarian cancer cells. In addition, miRNA-4478 targets Fus to inhibit autophagy, thereby sensitizing ovarian cancer cells to radiation [69].

Zhang et al. found that miRNA-214 was significantly upregulated in ovarian cancer tissues and radioresistant ovarian cancer cell lines. Transfection of miRNA-214 agomir in radiosensitive ovarian cancer cell lines increased their resistance to ionizing radiation, whereas transfection of miRNA-214 antagomir in radioresistant ovarian cancer cell lines sensitized them to ionizing radiation again. And miRNA-214 was also found to effectively promote tumor radioresistance in xenograft animal experiments. Protein blotting and real-time fluorescence quantitative PCR showed that miRNA-214 negatively regulated PTEN in radioresistant ovarian cancer cell lines and ovarian cancer tissues. a series of experimental demonstrations led to the conclusion that miRNA-214 promotes radioresistance in ovarian cancer by directly targeting PTEN [105].

Zhao et al. established miRNA-210 overexpression and underexpression ovarian cancer cell models by cell transfection, treated the cells with different doses of ionizing radiation, and then detected the cell proliferation activity. Following that, the expression of apoptosis-related proteins was detected by protein blotting. The experimental results showed that miRNA-210 could reduce the sensitivity of ovarian cancer cells to radiotherapy by inhibiting apoptosis, which may be a potential target for the treatment of ovarian tumors [106].

### Response to chemotherapy

Surgery and platinum-based chemotherapy are the main treatments for ovarian cancer, and 70% of ovarian cancer patients present with metastases at the time of diagnosis, 80–90% of whom have missed surgery [107]. Therefore, cisplatin (DDP)-based chemotherapy is an important approach for the treatment of ovarian cancer. However, treated ovarian cancer cells often develop resistance to DDP, making the overall efficacy of DDP unsatisfactory in clinical practice. Patients with drug-resistant ovarian cancer have a poorer prognosis and a low response rate to further chemotherapy. Therefore, efforts to overcome drug resistance in ovarian cancer are a dynamic and vast field [108]. Autophagy-related miRNAs have been reported to affect the sensitivity of cancer cells to anticancer drugs, as shown in Table 3. Kazmierczak D by miRNA microarray of two cisplatin and two paclitaxel-resistant A2780 cells, observed changes in miRNA expression levels that 46 changes in miRNA expression may be associated with drug resistance. Interestingly, the same miRNA expression changes were observed in two cisplatin-resistant cell lines and two paclitaxel-resistant cell lines. Upon target analysis, it was shown that important resistance genes such as protein Tyrosine Phosphatase Receptor Type K (PTPRK), Semaphorin 3A (SEMA3A), or the ATP-binding cassette subfamily B member 1 gene (ABCB1) expression can also be regulated by miRNAs [109]. Not coincidentally, autophagy is also closely related to chemoresistance of tumors, and its self-protective capacity increases the resistance of cancer cells to chemotherapeutic agents [110]. The resistance of tumor cells to chemotherapeutic drugs is too complex, and due to the complexity of DDP resistance mechanism, the treatment of ovarian cancer needs to improve the specificity and effectiveness, and multi-pathway combination therapy is undoubtedly one of the best options [111]. Examples include the combination of multiple antitumor drugs [112], new dosage form design to overcome tumor resistance [113] and active product reversal of tumor resistance [114].

**Table 3:** The impact of autophagy-related miRNAs on ovarian cancer chemotherapy and radiotherapy.

Effect on autophagy	Name	miRNA expression regulation	Resistance to treatment	Effect on radiotherapy/chemotherapy	Tested cell line (tissue origin)	Ref.
Inhibition	miRNA-125b	Upregulated	Cisplatin	Sensitized	COC1/DDP	[118]
Inhibition	miRNA-133a	Upregulated	Cisplatin	Sensitized	A2780/DDP and SKOV3/DDP	[116]
Inhibition	miRNA-219-5p	Upregulated	Cisplatin	Sensitized	A2780/DDP and SKOV3/DDP	[79]
Inhibition	miRNA-299	Upregulated	Radiotherapy	Sensitized	Caov3	[148]
Inhibition	miRNA-3127-5p	Downregulated	paclitaxel	Sensitized	SKOV3, A2780, SKOV3/PTX and A2780/PTX	[78]
Inhibition	miRNA-4478	Upregulated	Radiotherapy	Sensitized	ES-2 and SKOV3	[69]
Inhibition	miRNA-506-3p	Upregulated	carboplatin	Sensitized	SKOV3/CBP and A2780 / CBP	[76]
Inhibition	miRNA-1301	Upregulated	Cisplatin	Sensitized	SKOV3/DDP	[133]
Inhibition	miRNA-29c-3p	Upregulated	Cisplatin	Sensitized	SKOV3/DDP A2780/DDP	[135]
Inhibition	miRNA-29b-3p	Downregulate	paclitaxel	Sensitized	SKOV3/PTX and A2780/PTX	[129]
Inhibition	miRNA-199a	Downregulate	Cisplatin	Sensitized	SKOV3/DDP	[134]
Inhibition	miRNA-200c	Upregulated	Olaparib	Sensitized	SKOV3/Olaparib	[136]
Inhibition	miRNA-588	Downregulate	Radiotherapy	Sensitized	SKOV3 and A2780	[149]
Inhibition	miRNA-374a	Downregulate	Cisplatin	Sensitized	A2780/DDP	[150]
Inhibition	miRNA-770-5p	Upregulated	Cisplatin	Sensitized	A2780/DDP and SKOV3 / DDP	[151]
Inhibition	miRNA-1299	Upregulated	paclitaxel	Sensitized	SKOV3/PTX	[152]
Activation	miRNA-181d	Upregulated	Cisplatin	Resistant	A2780/DDP	[153]
Activation	miRNA-210	Upregulated	Radiotherapy	Resistant	OVCAR3 and SKOV3	[106]
Activation	miRNA-214	Upregulated	Radiotherapy	Resistant	SKOV3 and IOSE80	[105]
Activation	miRNA-27a	Upregulated	paclitaxel	Resistant	A2780、HO8910、OVCAR3 and PEO-1	[154]
Activation	miRNA-194-5p	Upregulated	Cisplatin	Resistant	A2780/DDP and COC1/DDP	[155]

In recent years, a growing number of studies have shown that autophagy is associated with chemoresistance in ovarian cancer and that miRNAs can reverse tumor cell resistance to cisplatin through the regulation of autophagy. Cai et al. showed that miRNA-30a inhibits autophagy and reduces chemoresistance to DDP in ovarian cancer cells by inhibiting activation of the TGF-β/Smad4 pathway, thereby improving the efficacy of DDP Clinical efficacy of ovarian cancer treatment [115]. Zhou et al. found that miRNA-133a can directly target YES3 in combination with its 3′UTR region and that the miRNA-133a/YES1 axis may regulate cisplatin sensitivity in ovarian cancer via autophagy, providing the possibility that the miRNA-133a/YES1/autophagy axis may serve as a novel diagnostic biomarker and potential gene therapy target for ovarian cancer chemotherapy [116]. Song et al. miRNA-219-5p reduces cisplatin resistance in ovarian cancer cells by regulating HMGA2, followed by Wnt/β-linked protein signaling (catenin signaling) and autophagy [117]. Thus, the role of miRNAs in tumor cell resistance suggests that targeting miRNA-mediated autophagy is a potential strategy to mitigate drug resistance in patients with advanced tumors.

For example, miRNA-20a-5p was shown to inhibit autophagy and cisplatin resistance in ovarian cancer through DNMT3B-mediated DNA methylation of RBP1 [67]. Similarly, miRNA-125b and miRNA-133a, when overexpressed, promote the autophagic process in drug-resistant tumor cells thereby reducing cisplatin resistance in tumor cells [116, 118]. In addition, miRNA-3127-5p and miRNA-506-3p have been shown to modulate autophagy in drug-resistant tumor cells to enhance chemosensitivity to paclitaxel and carboplatin, respectively [76, 78].

However, on the other hand, a large number of studies have found that aberrant expression of miRNAs also causes inhibition of apoptosis, which leads to chemoresistance. For example, Xu et al. showed that miRNA-149-5p was demonstrated to be significantly expressed in cisplatin-resistant ovarian cancer cells, and silencing of miRNA-149-5p enhanced the chemosensitivity of ovarian cancer cells to cisplatin in vitro and in vivo. Conversely, the upregulation of miRNA-149-5p exacerbated chemoresistance in ovarian cancer cells [119]. Similarly, in an ovarian tumor xenograft mouse model, overexpression of miRNA-204 enhances cisplatin resistance, whereas decreased expression of miRNA-630 increases paclitaxel sensitivity [120]. Wang et al. found that cisplatin-resistant epithelial ovarian cancer cells were enriched with miRNA-98-5p, a member of the let-7 family, compared to cisplatin-sensitive cells, and could promote cisplatin resistance in epithelial ovarian cancer cells [121].

Indeed, autophagy-associated miRNAs are involved in the regulation of tumor resistance of ovarian cancer by various antitumor drugs. With the in-depth elucidation of the mechanisms associated with autophagy-related miRNAs and ovarian cancer chemoresistance, there will be more and more autophagy-related miRNAs that may become anticancer targets.

### Preclinical or clinical trials

Although a large number of literatures have reported that miRNA-mediated autophagy has great potential in ovarian cancer treatment, it has not advanced to the clinical trial stage. Currently, the application of miRNAs in ovarian cancer is mainly focused on the early screening and diagnosis of ovarian cancer.

Liquid biopsy techniques represented by the detection of biomarkers in extracted exosomes are potential tools to address noninvasive and early screening methods to improve the diagnosis of patients with ovarian cancer [122]. Exosomes are tiny vesicles secreted by cells with a unique lipid bilayer membrane structure that protects the internal contents (e.g., mRNA and miRNA) well from degradation [123]. Thus, exosomes are more stable than other biomarkers presented in plasma. An increasing number of cancer-derived exosomal cyclins have been identified, and their expression levels are correlated with tumor progression. Due to their abundance, stability, ease of detection, and specific expression pattern in tumors, these exosomes are considered as new potential biomarkers for tumor diagnosis and prognosis. Zhu et al. used exosome liquid biopsy technique to detect the expression levels of four genes, miRNA-205, CA125, HE4, and TCF21, in plasma exosomes of ovarian cancer patients. Plasma exosomal miRNA-205 was found to have significant advantages in the diagnosis of ovarian cancer, and when combined with traditional serum tumor biomarkers, it could improve the diagnostic efficiency of ovarian cancer. In addition, plasma exosomal miRNA-205 levels were associated with ovarian cancer staging and lymph node metastasis, providing a valuable reference for early diagnosis and prognostic assessment of ovarian cancer patients [124].

Exosomal miRNAs have been associated with ovarian cancer progression and drug resistance and have been studied in great depth [125]. However, we are still far from fully understanding their biological functions to translate for clinical use. Further studies are needed to determine the function of exosomes in vivo. The development of a rapid, standardized, and efficient method for exosome isolation is also a pressing need for future ovarian cancer research as it is a challenge to isolate large amounts of pure exosomes in a standardized way for the comparison of studies.

## Conclusion and prospection

miRNAs are involved in the regulation of cellular physiological processes through a variety of signaling pathways, which also include the regulation of tumor cells through the regulation of cellular autophagy. More and more studies have shown that miRNA-mediated autophagy plays a key role in several aspects of ovarian cancer and exhibits a significant regulatory effect on ovarian cancer cells. Interestingly, this regulatory effect exhibits a dual nature. On the one hand, miRNA expression can inhibit ovarian cancer progression by regulating the autophagy process to promote apoptosis of ovarian cancer cells and increase their sensitivity to radiotherapy and chemotherapy. On the other hand, aberrant miRNA expression may also lead to ovarian cell resistance to radiotherapy and chemotherapy, and enhance the proliferation, invasion and metastasis of ovarian cancer cells.

Nevertheless, the clinical application of miRNA agents or miRNA mimics as novel ovarian cancer therapeutic agents needs to overcome many challenges. First, an in-depth study of the large number of miRNAs associated with the course of ovarian cancer, as well as their effects and mechanisms of action in tumor tissues and other tissues, is required. Second, miRNA agents or miRNA mimics with targeted and specific modes of action must be designed to improve their effectiveness in tumor therapy. It is precisely the fact that there are still many unresolved scientific issues that have hindered the clinical development of miRNAs for the treatment of ovarian cancer by modulating autophagy. However, it is now known through many means that the expression levels of many relevant miRNAs in the ovarian cancer process can be used as an important means of judging the ovarian cancer process. Clinical trials have been initiated to detect the miRNA levels in extracted exosomes for noninvasive and early ovarian cancer screening, and it is believed that in the near future, more and more miRNAs will be discovered and applied to ovarian cancer or other tumor cells. early screening and tumor progression analysis.

In conclusion, miRNAs can regulate autophagy to promote or delay ovarian cancer progression, which provides a basis for miRNAs to be used as therapeutic targets for ovarian cancer. However, no clinical studies have been reported on therapeutic drugs based on miRNAs regulating autophagy to regulate the progression of ovarian cancer, and therefore further in-depth studies on this mechanism are needed. The changes in autophagy of tumor cells also occur during the progression of ovarian cancer, which in turn causes alterations in miRNA expression. Some of these miRNAs are correlated with the progression of ovarian cancer. Therefore, early screening and progression assessment of ovarian cancer can also be performed by analyzing the expression levels of specific miRNAs in patients. In summary, miRNA-mediated autophagy plays a dual role in ovarian cancer and has great potential for diagnosis, treatment and prognostic assessment of ovarian cancer. However, to fully realize this potential, further in-depth research in this area is needed, as well as the translation of research results into clinical practice.
